# Within-Person Links Between Stress and Repetitive Thinking Intensify With Age

**DOI:** 10.1093/geroni/igaf122.4233

**Published:** 2025-12-31

**Authors:** Julie Kircher, Christopher Engeland, Dakota Witzel, Sarah Lipitz, Jennifer Graham-Engeland

**Affiliations:** The Pennsylvania State University, University Park, Pennsylvania, United States; The Pennsylvania State University, University Park, Pennsylvania, United States; South Dakota State University, Brookings, South Dakota, United States; The Pennsylvania State University, University Park, Pennsylvania, United States; The Pennsylvania State University, University Park, Pennsylvania, United States

## Abstract

Perceived stress and unconstructive repetitive thinking (URT) – persistent, negative thought patterns – are bidirectionally linked, often following a pattern in which stressors trigger URT that heightens perceived stress. These associations may be particularly strong in midlife, when exposure to and diversity of stressors are greater than in older adulthood. This suggests that older age may be a source of resilience; however, the moderating effect of age on associations between daily stress and URT remains largely untested. Using ecological momentary assessment, we examined how momentary perceived stress relates to URT and whether age affects this relationship. We hypothesized that older adults would report lower URT during moments when they also reported higher than usual perceived stress (compared to their own averages). Data came from a longitudinal measurement burst study, in which participants completed five assessments every day for 14 days. The sample, recruited via systematic probability from the Bronx NY, included 235 community-dwelling adults (Mage=45.99, Range=25–65). Multilevel models revealed that during moments of higher than usual perceived stress, momentary reports of URT were also higher (γ = 0.0344, SE = 0.0010, t(213)=34.18, p<.001). Age significantly modified the momentary perceived stress and URT association (γ = 0.0002, SE = 0.0001, t(213)=2.24, p=.025), such that the strength of this association increased with age. Simple slopes showed significant positive associations (p<.0001) across all age groups: (-1 SD/∼35 years) b = 0.032, (0 SD/∼46years) b = 0.034, (+1 SD/∼57years) b = 0.036. These findings highlight the importance of examining real-time links between URT and perceived stress to inform future work and, ultimately perhaps, personalized interventions for older adults.

